# Nephrotoxicity as a cause of acute kidney injury in children

**DOI:** 10.1007/s00467-007-0721-x

**Published:** 2008-12-01

**Authors:** Ludwig Patzer

**Affiliations:** Children’s Hospital St. Elisabeth and St. Barbara, Mauerstrasse 5, 06110 Halle/S., Germany

**Keywords:** Renal side effects

## Abstract

Many different drugs and agents may cause nephrotoxic acute kidney injury (AKI) in children. Predisposing factors such as age, pharmacogenetics, underlying disease, the dosage of the toxin, and concomitant medication determine and influence the severity of nephrotoxic insult. In childhood AKI, incidence, prevalence, and etiology are not well defined. Pediatric retrospective studies have reported incidences of AKI in pediatric intensive care units (PICU) of between 8% and 30%. It is widely recognized that neonates have higher rates of AKI, especially following cardiac surgery, severe asphyxia, or premature birth. The only two prospective studies in children found incidence rates of 4.5% and 2.5% of AKI in children admitted to PICU, respectively. Nephrotoxic drugs account for about 16% of all AKIs most commonly associated with AKI in older children and adolescents. Nonsteroidal anti-inflammatory drugs (NSAIDs), antibiotics, amphotericin B, antiviral agents, angiotensin-converting enzyme (ACE) inhibitors, calcineurin inhibitors, radiocontrast media, and cytostatics are the most important drugs to indicate AKI as significant risk factor in children. Direct pathophysiological mechanisms of nephrotoxicity include constriction of intrarenal vessels, acute tubular necrosis, acute interstitial nephritis, and—more infrequently—tubular obstruction. Furthermore, AKI may also be caused indirectly by rhabdomyolysis. Frequent therapeutic measures consist of avoiding dehydration and concomitant nephrotoxic medication, especially in children with preexisting impaired renal function.

## Introduction

Many different drugs and agents are currently being taken into consideration as the causality of nephrotoxic acute kidney injury (AKI) in children. Predisposing factors such as age, pharmacogenetics, underlying disease, dosage of the toxin, and concomitant medication determine and influence the severity of nephrotoxic insult. The culprit toxins are predominantly drugs, but exogenous (ethylene glycol, methylene) and endogenous (hemoglobin, myoglobin) substances and toxins from animals play a role as well. Throughout this teaching article, incidence, pathophysiological mechanisms, and treatment options are discussed in general followed by characteristics of problematic drugs. Because of the paucity of multicenter studies exploring AKI in children, previously conceived literature of AKI in adults ought to be considered.

Tumor lysis syndrome is not discussed. However, calcineurin inhibitor toxicity is briefly mentioned, as it has been respectively inclusive to the topic throughout multiple publications [1–8].

## Definition and incidence

Most authors define AKI as a sudden decline in glomerular filtration rate (GFR) mirrored by doublings of serum creatinine and azotemia. Because a precise clinical definition remains elusive, studies comparing epidemiology and outcome can be problematic (see Mehta et al. [9] for review). For oncological patients being treated with cytotoxic drugs, fractionated total body irradiation, and stem cell transplantation, AKI is due to multiple risk factors, with nephrotoxicity being one of the most significant. In this group of patients, a regimen-related toxicity score, as proposed by Bearman et al. [10], is often used. This score is defined as follows: grade 1—an increase in creatinine up to twice the baseline; grade 2—an increase in creatinine above twice the baseline but not requiring dialysis; grade 3—renal replacement therapy required; grade 4—fatal toxicity.

In adults, the overall incidence of AKI was found to be 209 per million population (0.02%). This figure was most likely generated by hypoxic/ischemic and nephrotoxic insults [11, 12]. Other studies report incidence rates of between 7% and 25% among critically ill adults [13–16]. This broad range is partly due to the many different coexisting definitions of AKI currently used, as mentioned above.

Community-based statistics estimate the incidence of AKI attributed to drug nephrotoxicity as being between 0% and 7% [17, 18] and the incidence of in-hospital AKI attributed to drug nephrotoxicity in adults at about 20% of all AKI [19–23]. Antibiotics (3–11%), angiotensin-converting enzyme (ACE) inhibitors (0.5–7%), NSAIDs (3–22%), and contrast media (2–12%) were noted as the most recurrent offenders. Depending on the publication date of the statistics, an increase in ACE inhibitors and a decrease in contrast media as causing agents was found during recent years (see de Broe et al. [24] for details). The trend in claiming higher frequencies of NSAIDs and ACE inhibitors as causes for drug-induced AKI was confirmed by a survey in 2001 by Ronco et al. [25]. It has been observed that hospital-acquired AKI is usually associated with one of three renal insults: a prerenal event, exposure to nephrotoxins, or sepsis [11]. Nephrotoxins, alone or in combination, contribute to at least 25% of all cases of hospital-acquired AKI [26]. In patients treated for oncological diseases, AKI was found to be between 0% and 40%, depending on the cytotoxic regimen used [10, 27–35].

In childhood AKI, incidence, prevalence, and etiology are not well defined. Pediatric retrospective studies have reported incidences of AKI in pediatric intensive care units (PICU) of between 8% and 30% [36–39]. It is widely recognized that neonates have higher rates of AKI, especially following cardiac surgery, severe asphyxia, or premature birth [37, 40–44]. The only two prospective studies in children admitted to PICU found AKI incidence rates of 4.5% and 2.5%, respectively [45, 46]. In the study of Bailey et al. [45] (excluding neonates), the most common admission diagnoses in AKI were hemolytic uremic syndrome (18.2%), oncologic pathologies (18.2%), and cardiac surgery (11.4%). Although these authors could demonstrate by univariate analysis that nephrotoxic drugs were used more often in children with AKI, they could not indicate nephrotoxic drugs as a risk factor for AKI by multivariate analysis. A 2005 report from Houston, Texas, USA, stated the most common causes of AKI in hospitalized children were renal ischemia (21%), pharmacologic agents (16%), and sepsis (11%). Primary renal disease accounted for only 7% of all cases [38]. Furthermore, this study associated nephrotoxic medications with AKI in primarily older children and adolescents. The frequency of AKI in pediatric patients following hematopoietic stem cell transplantation (HSCT) is between 6% and 50%, of whom 5–10% require renal replacement therapy [47–50].

## Drug-induced AKI: mechanisms and clinical significance

Table 1 illustrates a classification of drugs known to cause AKI regarding pathophysiologic mechanisms (from Porter et al. in [24]). The following text reviews in more depth the aspects of the most common substances at risk for causing AKI in children.

**Table 1:** Classification of various drugs based on pathophysiologic categories of acute kidney injury

Pathophysiology	Drugs known to cause acute kidney injury
Prerenal failure	NSAIDs, ACE inhibitors, cyclosporine A (CyA), norepinephrine, AT2-receptor antagonists, diuretics, interleukins, cocaine, mitomycin C, tacrolimus, estrogen, quinine
Acute tubular necrosis	Antibiotics: aminoglycosides, cephalosporins, amphotericin B, rifampicin, vancomycin, foscarnet, pentamidine
	NSAIDs, glaphenin, contrast media, acetaminophen, CyA, cisplatinum, i.v. immunoglobulin, dextran, maltose, sucrose, mannitol, heavy metals
Acute interstitial nephritis	Antibiotics: ciprofloxacin, methicillin, penicillin G, ampicillin, cephalosporins, oxacillin, rifampicin
	NSAIDs, glaphenin, acetylsalicylic acid (ASA), fenoprofen, naproxen, phenylbutazone, piroxicam, tolmetin, zomepirac, contrast media, sulfonamides, thiazides, phenytoin, furosemide, allopurinol, cimetidine, omeprazole, phenindione
Tubular obstruction	Sulfonamides, methotrexate, methoxyflurane, glaphenin, triamterene, acyclovir, ethylene glycol, protease inhibitors
Hypersensitivity angiitis	Penicillin G, ampicillin, sulfonamides
Thrombotic microangiopathy	Mitomycin C, CyA, oral contraceptives

From reference [24]

### NSAIDs

NSAIDs possess antipyretic, analgesic, and anti-inflammatory effects. They are frequently used in children and have numerous therapeutic indications; for example, in the treatment of fever, postoperative pain, and inflammatory disorders such as juvenile idiopathic arthritis and Kawasaki disease. Their major mechanism of action is through inhibition of prostaglandin biosynthesis by blockade of cyclooxygenase (COX). In euvolemic states, prostaglandins have a negligible effect on renal hemodynamics. In states of volume depletion, there is upregulation of the renin–angiotensin system as well as increased catecholamine release that, in addition to producing renal vasoconstriction, stimulates renal prostaglandin production. These prostaglandins counteract the vasoconstrictor effect by decreasing preglomerular resistance and thereby maintaining renal perfusion and glomerular filtration. This protective effect is inhibited by the blockade of prostaglandin synthesis by NSAIDs. The disposition of most NSAIDs has been mainly studied in infants older than 2 years of age [51]. Next to paracetamol, it is likely that NSAIDs are the most often prescribed substances in pediatrics today.

Although AKI is a well-known adverse effect of these drugs, the incidence of nephrotoxicity due to NSAIDs, especially in children, is unknown. It is probable that these substances carry a good risk–benefit ratio when taken under adequate supervision. However, when taken continually by the elderly or individuals with comorbid conditions (dehydrated children), the frequency of adverse reaction rises dramatically.

The vast majority of healthy children who ingest therapeutic doses of NSAIDs for a limited duration tolerate them without any significant adverse effects. However, the risk of renal toxicity potentially increases in situations where there is stimulation of the renin–angiotensin system; for example, with volume depletion or preexisting chronic renal disease. Some case reports of AKI following NSAID administration have been published. In most of these cases, dehydration played an important role [52–54]. But even without concomitant treatment or dehydration, AKI following the intake of naproxen, diclofenac, ibuprofen, dipyrone, ketorolac, and paracetamol has become documented and published [55, 56]. The onset of AKI after the intake of medication ranged from 1 to 5 days. Oligo-/anuric and nonoliguric AKI has been documented and published. Ultrasound showed large kidneys with hyperechoic parenchyma, and urine analysis revealed no specific changes that would allow distinction from other causes of AKI. Though kidney biopsies in two out of three patients showed normal results, one out of three revealed interstitial inflammation. All documented patients showed good recovery of renal function. Also, AKI in three children treated with the COX-2 inhibitor rofecoxib has been recently published. Evidently, all children were managed without renal replacement therapy. However, a renal biopsy done in one child showed acute interstitial nephritis [57]. The COX-2 inhibitor celecoxib has been reportedly responsible for 24% of adult AKI [58, 59].

In neonates the therapeutic and/or preventive use of ibuprofen and indomethacin for closure of patent ductus arteriosus (PDA) in preterm and/or low birth weight infants was analyzed in a Cochrane review. It showed that ibuprofen reduced the risk of oliguria. It also demonstrated that a prolonged course of indomethacin was associated with a decreased incidence of renal function impairment, as evidenced by a lower proportion of infants having diminished urine output [60–62]. Currently, several cases of severe and sometimes irreversible renal insufficiency have been documented in neonates exposed to indomethacin prenatally or in the first days of life for treatment of PDA [63, 64].

In adults treated with Mesalazine because of inflammatory bowel disease, nephrotoxicity is reportedly evident in the treatment of one out of every 150 adult patients. Although more than 30 cases in adults have been described in the literature, only three cases of pediatric patients younger than 16 years have been published (see Biervliet et al. [65] for details). There is no relation to dosage or type of Mesalazine used; the underlying mechanism was interstitial nephritis leading to acute or chronic renal failure.

In summary, NSAIDs- and COX-2-inhibitor-induced renal failure in children is rare. However, if found, it is usually reversible after drug discontinuation. Caution should be taken when NSAIDs are administered to individuals with dehydration, preexisting renal problems, or in combination with other potentially nephrotoxic drugs.

### Antibiotics, amphotericin B, and antiviral agents

#### Aminoglycosides and vancomycin

Nephrotoxicity is a well known side effect of all aminoglycosides. Acute tubular necrosis is the most frequent mechanism of toxicity, but different patterns of tubular dysfunction have been described as well [66]. Aminoglycosides are not metabolized and eliminated by glomerular filtration and are partially absorbed into proximal tubular cells by megalin-dependent endocytosis. This uptake is saturable. In the lysosomes, aminoglycosides bind to phospholipids, leading to inhibition of phospholipase activity and consequently to phospholipid accumulation within the proximal tubular cell. Although the exact mechanism of tubular cell death caused by phospholipid accumulation has never been clearly established, there is a good correlation between the extent of phospholipidosis and toxicity induced by aminoglycosides. Furthermore, aminoglycosides induce alterations at the basolateral membrane and mitochondria, and they seem to enhance the generation of free radicals and lipid peroxidation in renal cortex. They also may alter glomerular function through mesangial cell contraction and may stimulate mesangial cell proliferation and apoptosis with no overt net change in total cell number [67].

Thirty years ago, the incidence of significant reduction of GFR present in seriously ill adult patients was found to be between 5% and 9% [68] and to rise with advanced age [69–71]. Neonates are at higher risk for aminoglycoside-induced nephrotoxicity. It is published that drug dosage, timing, duration of administration, dosing interval, preexisting renal disease, volume depletion, hepatic dysfunction, sepsis, and concomitant nephrotoxic and diuretic drugs may increase nephrotoxicity of aminoglycosides, but some of the published data are conflicting [24, 72]. It is especially not clear whether dosing only once daily decreases the risk of nephrotoxicity. This question was addressed in children by Uijtendaal et al. [73] in a randomized controlled trial (RCT) that found equal effectiveness and nephrotoxicity of gentamicin. In contrast in a meta-analysis of children with cystic fibrosis, Smyth et al. [74] found decreased toxicity in once-a-day treatment. As published by Verpooten et al., neither monitoring drug serum peak levels nor adjusting dosage (pharmacokinetic dosing) had any effect on nephrotoxicity [75].

A recent Cochrane analysis by Rao et al. [76] found that in neonates, there is insufficient evidence from available RCTs to conclude whether a regimen of once a day or multiple doses a day of gentamicin is superior in treating proven neonatal sepsis. However, data suggests that pharmacokinetic properties of a once-a-day regimen are superior to multiple doses a day because it achieves higher peak levels while avoiding toxic trough levels without change in nephrotoxicity or auditory toxicity.

Beta (β)-lactam antibiotics comprise penicillins, cephalosporins, monobactams, clavulanic acid, and carbapenems. Cephalosporins and carbapenems have been associated with nephrotoxicity in humans [77, 78]. Nephrotoxic β-lactam antibiotics cause acute proximal tubular necrosis with or without oliguria. Significant renal toxicity, which has been rare with penicillins and uncommon with cephalosporins, is a greater risk with the penems. The true incidence of β-lactam-antibiotics-induced AKI is unknown, as is how often it occurs when the antibiotic is used as monotherapy or in combination with other nephrotoxic drugs. Mechanisms of injury include: (1) transport into the tubular cell (mainly through the basolateral organic anion secretory carrier), (2) acylation of target proteins causing respiratory toxicity by inactivation of mitochondrial anionic substrate carriers, and (3) lipid peroxidation [77]. Renal toxicity from β-lactams may also result from hypersensitivity reactions. In these cases, glucocorticoids have been used, as they may improve recovery [79]. Cases of ceftriaxone-induced hemolysis and nephrolithiasis complicated by AKI have also been published [80–82]. In preterm neonates, the administration of ceftazidime was associated with a greater risk of acute kidney injury [83]. Ampicillin and aztreonam combination therapy in neonates showed a lower renal toxicity than in the group with concurrent administration of oxacillin and amikacin [84]. There are no specific therapeutic options in β-lactam-induced AKI; hemolysis and postrenal failure by stones need to be excluded, and there is no evidence that hemodialysis will improve recovery. “Judicious use of the drugs” [85] has become the most important approach to decreasing β-lactam nephrotoxicity, especially in neonates, where accurate determination of dosage is required for drugs with low therapeutic index and in patients with renal injury.

Macrolide antibiotics such as erythromycin, clarithromycin, azithromycin, and dirithromycin may indirectly cause AKI via enhancing calcineurin levels by inhibiting cytochrome P-450 isoenzyme CYP 3A4 [86]. This needs to be considered, especially in transplanted or nephrotic patients on CyA or tacrolimus.

A number of additional anti-infective drugs that may cause AKI in children include sulfonamides, trimethoprim-sulfamethoxazole (TMP-SMX) and quinolones. Some of these are of interest because of their use in treating infectious complications in patients with HIV infections. Systematic evaluation of renal side effects of these substances in children are lacking, though aspects of nephrotoxicity in adults are discussed in detail by Vaamonde et al. [87].

Nephrotoxicity of amphotericin B has been a matter of research for a long time. Despite its significant renal side effects, amphotericin B is still the drug of choice in the treatment of severe systemic fungal infections, especially in immunocompromised children. Its nephrotoxicity and measures of prevention have been recently reviewed by Koren and coworkers [88, 89] and Fanos et al. [90], but studies in children remain scarce. In retrospective studies, nephrotoxicity was found in 20–80% of adults and children [91–95]. Of transplant patients receiving amphotericin B, AKI was found to be in 0.5% of heart and/or lung transplants and 31% of bone marrow transplants [96]. The two major hypotheses for the pathogenesis of amphotericin-B-induced AKI are: (1) direct effects of the drug on ergosterol in the epithelial cell membranes and (2) renal vasoconstriction due to increased vascular resistance. Typically, intravenous administration results in an acute decrease of GFR and transient oliguria followed by polyuria. Unlike most other drug-induced nephrotoxicity, most patients develop distal tubulopathy as well. The pharmacokinetics of amphotericin B in children appears to differ from that in adults because distribution volume is smaller and the clearance rate is faster. As elimination half-life appears to be inversely correlated with a patient’s age, individualized dosing based on drug monitoring has been recommended. But the age-dependent nephrotoxicity of amphotericin B has not yet been addressed by studies. Risk factors for amphotericin-induced AKI include cumulative dose, treatment duration and dosing schedule, concomitant therapy with diuretics or other nephrotoxic drugs, and impaired GFR at baseline. As shown by a number of RCTs in adults, the introduction of the lipid formulation of the drug (lipid complex, colloidal dispersion, and liposomal form) decreased nephrotoxicity enormously [97]. Goldman et al. [88] proposed guidelines for preventing amphotericin-induced nephrotoxicity by suggesting a saline infusion prior to administration (10–15 ml/kg body weight) and use of lipid formulations of amphotericin B in children with either known side effects to conventional amphotericin B, reduced GFR, or concomitant nephrotoxic medication.

Antiviral agents (acyclovir, valacyclovir, ganciclovir, cidofovir, foscarnet, amantadine, and ribavirin) have become increasingly available for clinical use, especially to treat HIV infections. AKI is a significant and potentially therapy-limiting side effect to some of them. Antiviral agents have also been known to cause irreversible renal damage in adults [98]. Antiviral drugs cause renal failure through a variety of mechanisms. Direct renal tubular toxicity has been found in the use of a number of these medications, with unique effects on kidney epithelial cells. These drugs include cidofovir, adefovir dipivoxil, tenofovir, and acyclovir. Additionally, crystal deposition in the kidneys may promote development of renal failure, as in the use of acyclovir and the protease inhibitor indinavir. However, it is unclear whether the pathogenesis of acyclovir-induced AKI reflects an obstructive uropathy from intratubular precipitation of acyclovir, a hemodynamic response, or an immunologic, toxic, or hypersensitivity reaction. Furthermore, these substances effect the tubular transporters as well as tubule cells directly [99]. The exact frequency of nephrotoxicity induced by antiviral drugs is difficult to determine and not exactly known. AKI by acyclovir infusion was found to be between 10% and 16% in adults and 11% in children [100]. A recent Brazilian study found AKI in 19.5% of the 41 children treated intravenously. Their creatinine levels peaked in a mean time of 7.1 days (ranging from 3 to 14 days). Recovery of renal function, evaluated by the decrease of creatinine levels, varied from 1 to 7 days (mean 3.6 days) [101]. Vomiero et al. [102] reported AKI in three out of 17 children treated with ceftriaxone and acyclovir combination therapy because of meningoencephalitis pointing out that the addition of a second nephrotoxic drug aggravated the extent of acyclovir’s renal injury. Risk factors again include volume contraction, preexisting renal insufficiency, high dose, or rapid bolus infusion. AKI in children caused by valacyclovir and ganciclovir has not been published.

Foscarnet causes a reversible decrease in GFR in 20–60% in adults [103–106]. Two mechanisms for renal damage have been reported: acute tubular necrosis and crystallization within glomerular capillaries with crescentic nephritis. Hydration at the time of administration is thought to reduce the incidence of AKI [72]. Foscarnet-induced AKI is usually reversible, though temporary dialysis may be required. Volume expansion was once again effective in reducing the incidence of foscarnet nephrotoxicity [107]. In an RCT by Reusser et al. [108] of foscarnet vs. ganciclovir in the preemptive therapy of cytomegalovirus infection after allogeneic stem cell transplantation, renal function impairment was observed in five (5%) patients on foscarnet vs. two (2%) patients on ganciclovir.

Published data regarding renal failure in antiretroviral agents is scarce, especially in children. Some patients have been reported with AKI attributed to ritonavir, but the risk of AKI in this group of drugs appears low.

### ACE inhibitors and angiotensin II receptor type 1 antagonists

The use of ACE inhibitors and angiotensin II receptor type 1 antagonists (AT2-receptor antagonists) has increased dramatically over the last decade [109–115]. ACE inhibitors are the best documented treatment for delaying progression of chronic nondiabetic renal diseases and nephropathy in patients with type 1 diabetes. However, some major concerns appear to restrict the widespread use of these drugs, including acute interstitial nephritis associated with ACE inhibitors and a fall of GFR in some risk groups [116, 117]. In this educative article, we briefly mention some aspects of AKI and ACE inhibitors and refer to some reviews about ACE inhibition in adults and children [114, 118].

The presence of AKI due to ACE-inhibitor-induced interstitial nephritis has been described in few adult cases, but an occurrence of this kind in children has yet to be documented [119]. In contrast, however, prerenal AKI in children with nephrotic syndrome, impaired renal function, hypertension, congestive heart failure, or postrenal transplant patients treated with ACE inhibitors alone or in combination with AT2-receptor antagonists are well known in pediatric renal (and cardiac) clinics and have been reported [116, 117, 120–122].

It is generally accepted to be true, though not exactly proven, that the renal hemodynamic effects of ACE inhibitors are due to the blockade of a renin–angiotensin system. In a significant percentage of renal patients (renal artery stenosis, end-stage renal disease) and in patients with congestive heart failure, especially when volume depleted (diuretics, nephrotic syndrome), the maintenance of GFR is highly dependent on angiotensin-II-mediated efferent vasoconstriction. In the case of instituting ACE inhibitors and/or AT2-receptor antagonist treatment, close surveillance of renal function and blood pressure is needed, at least during the first 2 weeks, because a decline in renal function may be transient. The treatment should be started at low doses then gradually increased. The concomitant use of NSAIDs should be avoided, because in these situations, the afferent vasoconstriction (by prostaglandin synthesis inhibition) in combination with efferent vasodilatation (due to ACE inhibition) may result in a marked decline of GFR. The sometimes inevitable combination of ACE inhibitors and calcineurin inhibitors needs close monitoring and expertise.

AKI has not been seen after the administration of other antihypertensive agents that do not interfere with the renin–angiotensin system.

### Calcineurin inhibitors

The therapeutic potential and renal side effects of CyA and tacrolimus are well known to pediatric nephrologists. The introduction of calcineurin inhibitors has improved the outcome of renal and other organ transplants dramatically, but cyclosporine-related nephrotoxicity has become a significant side effect to manage [8]. Indeed, nephrotoxicity is the most significant and limiting adverse effect caused by calcineurin inhibitors. This paper only discusses aspects of AKI related to calcineurin inhibitors. Acute nephrotoxicity is a hemodynamically mediated phenomenon characterized by the absence of permanent structural changes and reversibility with decrease or discontinuation of the offending or concomitant nephrotoxic (interacting) drug [123].

GFR impairment is caused by intrarenal vasoconstriction that induces a decrease in renal blood flow. A great deal of effort has been taken to unravel the mechanism of CyA-induced vasoconstriction. Some mechanisms and mediators have been found. The renin–angiotensin–aldosterone system is involved [124], as are endothelin (a very potent constrictor of intrarenal vessels), nitric oxide, prostaglandins, free radicals, sympathetic system, vasopressin, atrial natriuretic factor(s), and some further substances [123]. The clinical presentation of acute CyA toxicity may present as AKI, asymptomatic increase of serum creatinine, hemolytic uremic syndrome (HUS), or a delayed recovery of renal graft function after renal transplantation. The exact incidence of these forms are unknown. The most frequent is an asymptomatic increase of serum creatinine. Clinically relevant AKI is published in 10% to more than 50% of all patients in the early phase after nonrenal transplantation [125–129]. The exact incidence in the pediatric population is unknown, because AKI in these patients is generally multifactorial and seldom related exclusively to CyA.

Especially in renal transplant patients, nephrotoxicity caused by calcineurin inhibitors may be difficult to distinguish from transplant rejection. In this situation, a renal biopsy may be indicated. Otherwise, in extrarenal organ transplantation and nonrenal autoimmune disease patients, a creatinine rise is very likely to be caused by CyA. As mentioned above, interaction of concomitant drugs enhancing CyA levels via inhibition of cytochrome P450 system (especially macrolide antibiotics, calcium channel antagonists, and antifungal azoles) needs to be considered, as this combination can cause AKI. Drugs that decrease renal blood flow should also be avoided whenever possible. Especially in bone marrow transplantation (BMT), CyA can cause acute HUS, usually with severe AKI, carrying a poor prognosis.

The acute nephrotoxicity profile of tacrolimus is very similar to that of CyA, as it induces reversible functional changes with the same frequency and intensity [123]. There are still no satisfactory strategies and measures to prevent CyA- and tacrolimus-induced AKI in children. Studies on omega-3 fatty acids, cilastatin, and endothelin receptor antagonists have been published with clinically positive results in preventing GFR impairment, but none of them are used routinely in clinical practice. One would recommend regular monitoring of blood levels, caution with concomitant medication, and avoidance of dehydration. Nevertheless, despite all these measures, CyA and tacrolimus still carry a significant risk of acute nephrotoxicity in patients who suffer from severe diseases. New substances will probably allow protocols without calcineurin inhibitors, minimizing the risk of drug toxicity and improving allograft and patient survival [130].

### Radiocontrast media

The incidence of AKI after administration of tri-iodinated radiocontrast media has been found to be between 1% and 20% in adults [131]. Incidence in children is unknown. As the new nonionic contrast media are less nephrotoxic, one would assume that the frequency of AKI related to contrast media has dropped over the last decade, but this is counterbalanced by an increase in the use of radiographic contrast investigations in an older age group. Furthermore, nonionic contrast media were found without any benefit in patients with normal renal function [132, 133].

Radiographic contrast-agent-induced nephropathy is caused by vasoconstriction-mediated renal medullary ischemia and direct toxic damage to renal tubular epithelial cells. These effects may be partially mediated by the generation of reactive oxygen species. Most cases present as nonoliguric AKI, although oliguric renal failure is possible as well [72]. Maximum creatinine rise usually occurs 3–5 days after admission of contrast media. Urine analysis often reveals granular casts, tubular epithelial cells, and minimal proteinuria but may be entirely unremarkable in some cases. Usually, the fractional excretion of sodium is very low. Risk factors include baseline impairment of renal function, diabetes mellitus, congestive heart failure, higher doses of contrast media, and concurrent use of nephrotoxic drugs. Of all these risk factors, preexisting renal function impairment appears to be the single most important. Preventive measures include intravenous hydration and administration of acetylcysteine [134–136]. However, the administration of calcium antagonists, theophylline, atrial natriuretic peptide, mannitol, furosemide, and dopamine has not shown to be effective [137]. In adults, dialysis is infrequently required, and some degree of residual renal impairment has been reported in about 30% of affected adults.

### Cytostatics

Nephrotoxicity related to cytotoxic treatment in children with oncological diseases is well known and has been reviewed several times [27, 138–145]. Ifosfamide causes predominantly chronic tubulopathy, sometimes beginning months or years after therapy. AKI has seldom been reported. In a study by Skinner et al. [141], impairment of GFR was observed in 61 of 123 patients, but overt renal failure was not found. However, in studies by Rossi et al., no acute renal failure has been reported [146–147]. Higher total ifosfamide dosage correlated significantly with greater glomerular toxicity. In adults, AKI related to ifosfamide has been documented secondary to tubular necrosis with high-dose therapy (>5g/m^2^), particularly in patients concurrently treated with cisplatin [148, 149]. In children, AKI case reports of prerenal failure due to water and sodium loss caused by the tubulopathy have been published [150].

Cisplatin may induce both acute and chronic renal toxicity [151]. A progressive and persistent reduction in GFR may follow each successive treatment cycle. The precise mechanisms involved in cisplatin-induced renal failure have not been completely clarified, but mitochondrial dysfunction and apoptosis are likely to play an important role. Inflammatory mechanisms may contribute to toxin-induced acute kidney injury as well [152, 153]. Early clinical studies with cisplatin reported dose-related AKI in more than 70% of adult patients [154]. In a series of 22 children receiving cisplatin, Womer et al. described a GFR of less than 80 ml/min per 1.73 m^2^ in 18 patients [155]. Polyuria regularly follows administration of cisplatin and occurs in two distinct phases. The first is within the first 24–48 h after administration, urine osmolality drops but GFR remains stable. This stage of early polyuria usually reverses spontaneously. The second phase of polyuria is 72–96 h after cisplatin administration and is characterized by an increase in urine volume and a persistent reduction in the GFR [156]. In efforts to reduce renal toxicity associated with cisplatin, therapeutic interventions have been aimed at the reduced production and enhanced excretion of highly reactive metabolites. Infusion of mannitol and saline is the clinical intervention most commonly used. The effectiveness of these measures is somewhat illustrated by a randomized study in adults by Al Sarraf et al. [157], but this protective effect was lost during subsequent cycles. The use of hypertonic (3%) saline infusion as an effective renoprotective measure awaits a randomized study as well, but cisplatin should not be given to volume-depleted patients. Another potential angle is to take a patient’s GFR into account in dosing. This has already been proposed by some therapeutic protocols. Also with cisplatin, the concurrent administration of NSAIDs, aminoglycosides, or other nephrotoxic drugs should be avoided.

Recently, a liposomal formulation of cisplatin, Lipoplatin, was developed to reduce systemic toxicity of cisplatin. It is under clinical evaluation in adult medicine, and phase 1 and phase 2 studies showed no nephrotoxicity up to a dose of 125 mg/m^2^ every 14 days [158, 159]. However, data on effectiveness and toxicity in pediatric oncology are not yet available.

Acute kidney injury has also been reported following administration of high-dose methotrexate (MTX). The most commonly accepted mechanism for this drug-induced toxicity is the precipitation of MTX and its metabolites in the distal tubules, leading to obstructive uropathy and tubular necrosis. Urinary alkalization and hydration offer protection against MTX-induced renal dysfunction. In patients with MTX-induced AKI, charcoal hemoperfusion and sequential hemodialysis with high-flux dialyzers were able to achieve a significant clearance of MTX. More recently, carboxypeptidase-G(2) [CPDG(2)] (a recombinant bacterial enzyme that rapidly hydrolyzes MTX to inactive metabolites) has become available in the treatment of MTX-induced renal dysfunction. CPDG(2) administration has been well tolerated and results in consistent and rapid reductions in plasma MTX concentrations by a median of 98.7%. Early administration of CPDG(2) in combination with leucovorin may be beneficial for patients with MTX-induced renal dysfunction and significantly elevated plasma MTX concentrations [160].

The combination of nephrotoxic drugs in the setting of HSCT intensifies nephrotoxic probability. Renal aspects of this specific therapeutic maneuver have been reviewed elsewhere [138], so here we summarize the important aspects of AKI in this very specific group of patients. Incidence of AKI immediately after HSCT in pediatric patients appears to be between 25% and 50% [161], with 5–10% requiring renal replacement therapy. In a prospective study involving 89 children, AKI was found in 25% within 30 days after HSCT. Specific renal syndromes appear in the course of HSCT at different time intervals, revealing a similar pattern in children in comparison with adult patients [27, 162]. In both children and adults, impaired renal function associated with liver impairment (hepatorenal syndrome) is the most significant cause of AKI. Further causes for AKI in the early phases after HSCT are septicemia, hypotension, drug toxicity, and the use of amphotericin B. To assess AKI risk factors in our pediatric population, we carried out logistic regression analyses and found allogeneic HSCT, conditioning containing cyclophosphamide, and etoposide, septicemia, hyperbilirubinemia, venoocclusive disease, and the use of spironolactone significantly correlated with AKI. However, age, diagnosis, and TBI did not. Specific therapeutic approaches have not yet been known to lower frequency or to improve outcome so far.

A growing number of case reports have been published in adult medicine regarding the nephrotoxicity of bisphosphonates. With regard to zoledronate, the incidence of AKI has been found to be between 9% and 13%. The pathology showed diffuse tubular atrophy and injury without interstitial nephritis. Bisphosphonate-induced AKI in children remains undocumented [163, 164].

Whereas therapy with intravenous immunoglobulin has been known to carry a 1–15% risk of AKI in adults [165–167], only a few case reports in children have been published [168]. Presumable risk factors consist of an impaired GFR and age over 65 years.

### Natural toxins

Toxin-induced AKI by prescription of herbal and alternative medicines is uncommon in Europe but has become a common cause of mortality and morbidity in Africa (see Swanepoel et al. for review [169] and [170]). Animal nephrotoxin-induced AKI is also uncommon in Europe but well known to colleagues from Africa, South America, and Australia. Table 2 outlines a list of common biologic nephrotoxins produced by animals. Many of these toxins cause AKI by direct or indirect mechanisms. The main indirect mechanism is rhabdomyolysis [171]. Some direct mechanisms include acute tubular necrosis, acute interstitial nephritis, necrotizing arteritis, and sometimes extracapillary glomerulonephritis. The consumption of a raw fish gallbladder containing toxic ichthyogallotoxin has been known to cause AKI in children and has been reported. In this instance, therapeutical approaches are symptomatic, and sometimes, intensive care and renal replacement therapy may be required.

**Table 2:** Common biologic nephrotoxins produced by animals. Modified from Chesney and Jones [72]

Animal	Biologic nephrotoxins
Snake	Phospholipase A_2_, myotoxins, procoagulant-activating factors V and X
Spider	Sphingomyelinase D, neurotoxins
Bee	Melittin, phospholipase A_2_, mast-cell degranulation protein
Wasp	Antigen 5, mastoparans
Murine animals (carp, jellyfish, sea anemone)	Ichthyogallotoxin, cyprinol

## Diagnosis, treatment, and outcome

Clinical management and supportive therapy of children with suspected or established AKI caused by nephrotoxicity is not unlike other forms. A child’s clinical history may help reveal which drugs had been administered within the previous 5–7 days. Postrenal obstruction needs to be excluded by ultrasound, and infective or immunological causes need to be addressed by laboratory investigations. Hemolysis and rhabdomyolysis need to be excluded, especially in cases of dark urine (which is sometimes misinterpreted as macrohematuria).

It is of great importance to assess the exact status of hydration (fractional excretion of sodium, urine sodium, urine osmolality) and to decide whether a load of fluid or diuretics may be necessary. Correction of volume depletion and/or congestive heart failure and reversing diminished renal perfusion are of primary importance. Especially in patients with dehydration, special attention has to be paid to good fluid management and replacement to establish adequate renal blood flow and perfusion. The administration of renal-dose dopamine (1–3 µg/kg min) was shown to be ineffective on patient survival or outcome in both adults [172–174] and children [175]. There is no evidence from randomized trials to support the use of dopamine to prevent renal dysfunction in indomethacin-treated preterm infants [176].

Diuretics have also been used in patients with oliguric AKI to improve urine output, which may decrease the concentration of toxins and improve clearance of solute and medications. It should be noted, however, that the increase in urine flow provided by diuretic therapy does not reflect an improvement in GFR, nor has it been demonstrated to prevent or facilitate recovery from acute tubular necrosis [177]. In addition, children with AKI may deteriorate further with diuresis, particularly patients with prerenal or radiocontrast-induced AKI [178].

As children with nephrotoxic insults are more likely to suffer nonoliguric renal failure, serum chemistry needs to be supervised very carefully in high-risk children. It has also to be considered that in children with reduced muscle mass (oncological diseases), GFR is overestimated by assessment of serum creatinine (or Schwartz formula). These two aspects may delay the diagnosis of AKI or may result in GFR overestimation. When medications are prescribed in AKI, the mechanism of drug elimination, metabolic pathway, and possible further nephrotoxic effects must be considered and adjustments made for the degree of renal failure.

In case of suspected or definite nephrotoxic agents being the cause of AKI, drug withdrawal is the most important measure physicians can take, as avoidance of further possible nephrotoxic agents needs to be strictly considered. In all children, the basic principles of managing AKI are fluid maintenance, electrolyte homeostasis, and adequate nutrition. Only few patients will require specific treatment as acetylcysteine in radiocontrast nephropathy. In interstitial nephritis, corticosteroids may be given, but their benefits are not proven by RCTs.

Regarding indication and optimal choice of renal replacement therapies, we refer to published reviews and guidelines [179–184].

The mortality of children with AKI was found to be between 8% and 89% in retrospective studies [39, 40]. In prospective studies, Bailey et al. report a mortality rate of 29.6% in patients with AKI compared with 2.3% in patients without [45], and the Spanish study found a mortality rate of 36% in patients with AKI [46]. Recent data from Houston showed a 3- to 5-year survival rate of 79.9% of children with AKI who survived to hospital discharge [185]. Most deaths (68.5%) occurred within 12 months after initial hospitalization. Combining those who died during initial hospitalization and in the subsequent 3–5 years, the overall survival rate was 56.8% [185]. Renal survival in survivors was 91%. Among 29 patients assessed at 3–5 years, 59% had at least one sign of renal injury (microalbuminuria, a decreased GFR, hyperfiltration, or hypertension). In that study, AKI was caused by nephrotoxic agents in 14.3%. With regard to the fate of individuals who develop AKI caused by nephrotoxicity, several outcomes have been reported in adults. In most cases, the drug’s nephrotoxicity is partly or wholly reversible. However, some authors have suggested that as many as 50–60% of adults who suffer nephrotoxicity may retain an element of permanent renal damage [186, 187].

In the setting of HSCT, the doubling of serum creatinine is associated with a twofold increase in mortality in adults, and the need for dialysis predicts a mortality rate of 80–90% [30, 188]. In comparison with adults, AKI and renal replacement therapy were also associated with an increase in mortality [47, 50, 189]. On the other hand, the predictive value of transient AKI for renal function in long-term survivors remains controversial. In some studies, renal impairment during the early phase of HSCT was not predictive of later renal impairment [48, 190], whereas Kist-van Holthe et al. found correlations between high serum creatinine within 3 months after BMT and chronic renal failure 1 year after BMT [49] but only a weak predictive value of acute AKI for chronic renal failure [191].

## Conclusion

The true incidence of nephrotoxicity as a cause of AKI in children is unknown. In general, drugs are an infrequent cause of community-acquired AKI, especially in children. However, drugs and hypoxia are the leading etiologic factors for hospital-acquired AKI, and it remains a significant cause of morbidity and mortality in children. Despite advances in understanding the pathophysiology of AKI, little progress has been made in its treatment. The judicious use of all nephrotoxic drugs and their combinations, together with adequate hydration, are still the most important measures to take in the minimization of nephrotoxicity. Children with AKI caused by nephrotoxic agents have a significant risk for chronic renal injury.

**Questions** (answers appear following reference list)

1. Which one of the following statements is true of NSAIDs-induced AKI?
  A In most children with NSAIDs-induced AKI, dehydration played an important role.
  B NSAIDs-induced AKI is mostly caused by interstitial nephritis.
  C Mesalazine-induced AKI is always caused by interstitial nephritis.
  D Glucocorticoids play an important role in treatment of NSAIDs-induced AKI.
2. Which one of the following statements is true? Neonates:
  A Have higher rates of nephrotoxicity-induced AKI compared with older children.
  B Have generally a poor long-term prognosis in relation to nephrotoxicity-induced AKI.
  C Benefit from a “pharmacological dosing” of gentamicin regarding nephrotoxicity.
  D Benefit generally from ibuprofen compared with indomethacin regarding the closue of PDA.
3. Which one of the following answers is not true?
  A β-lactam antibiotics need tubular transport for nephrotoxicity.
  B Renal replacement therapy always shortens the time of renal insufficiency by removing toxins.
  C Ceftriaxone may induce AKI indirectly by hemolysis and nephrolithiasis.
  D Macrolide antibiotics may cause indirect AKI by enhancing calcineurin levels by inhibiting cytochrome P-450 isoenzymes.
4. Which one of the following answers is true?
  A ACE inhibitors are forbidden in children with steroid-resistant nephrotic syndrome because of their risk of AKI.
  B The concomitant use of NSAIDs and ACE inhibitors should be avoided.
  C AKI caused by ACE inhibitors is always characterized by interstitial nephritis.
  D Concomitant therapy with AT2-receptor antagonists enhances side effects of ACE inhibitors.
5. Which one of the following answers is not true?
  A Nephrotoxicity is the most significant and limiting adverse effect caused by calcineurin inhibitors.
  B The acute nephrotoxicity of CyA is a hemodynamically mediated phenomenon.
  C A kidney biopsy is often necessary in renal transplant children to distinguish between CyA nephrotoxicity and rejection.
  D CyA-induced HUS in BMT carries a rather good prognosis.

## Abbreviations

AKI: acute kidney injury
BMT: bone marrow transplantation
COX: cyclooxygenase
CyA: cyclosporine A
GFR: glomerular filtration rate
HSCT: hematopoietic stem cell transplantation
MTX: methotrexate
NSAIDs: nonsteroidal anti-inflammatory drugs
PDA: patent ductus arteriosus
PICU: pediatric intensive care unit
RCT: randomized controlled trial
